# *Schistosoma mansoni* Venom Allergen Like Proteins Present Differential Allergic Responses in a Murine Model of Airway Inflammation

**DOI:** 10.1371/journal.pntd.0001510

**Published:** 2012-02-07

**Authors:** Leonardo Paiva Farias, Dunia Rodrigues, Vinicius Cunna, Henrique Krambeck Rofatto, Eliana L. Faquim-Mauro, Luciana C. C. Leite

**Affiliations:** 1 Centro de Biotecnologia, Instituto Butantan, São Paulo, São Paulo, Brasil; 2 Laboratório de Imunopatologia, Instituto Butantan, São Paulo, São Paulo, Brasil; Cambridge University, United Kingdom

## Abstract

**Background:**

The *Schistosoma mansoni* Venom-Allergen-Like proteins (SmVALs) are members of the SCP/TAPS (Sperm-coating protein/Tpx-1/Ag5/PR-1/Sc7) protein superfamily, which may be important in the host-pathogen interaction. Some of these molecules were suggested by us and others as potential immunomodulators and vaccine candidates, due to their functional classification, expression profile and predicted localization. From a vaccine perspective, one of the concerns is the potential allergic effect of these molecules.

**Methodology/Principal Findings:**

Herein, we characterized the putative secreted proteins SmVAL4 and SmVAL26 and explored the mouse model of airway inflammation to investigate their potential allergenic properties. The respective recombinant proteins were obtained in the *Pichia pastoris* system and the purified proteins used to produce specific antibodies. SmVAL4 protein was revealed to be present only in the cercarial stage, increasing from 0–6 h in the secretions of newly transformed schistosomulum. SmVAL26 was identified only in the egg stage, mainly in the hatched eggs' fluid and also in the secretions of cultured eggs. Concerning the investigation of the allergic properties of these proteins in the mouse model of airway inflammation, SmVAL4 induced a significant increase in total cells in the bronchoalveolar lavage fluid, mostly due to an increase in eosinophils and macrophages, which correlated with increases in IgG1, IgE and IL-5, characterizing a typical allergic airway inflammation response. High titers of anaphylactic IgG1 were revealed by the Passive Cutaneous Anaphylactic (PCA) hypersensitivity assay. Additionally, in a more conventional protocol of immunization for vaccine trials, rSmVAL4 still induced high levels of IgG1 and IgE.

**Conclusions:**

Our results suggest that members of the SmVAL family do present allergic properties; however, this varies significantly and therefore should be considered in the design of a schistosomiasis vaccine. Additionally, the murine model of airway inflammation proved to be useful in the investigation of allergic properties of potential vaccine candidates.

## Author Summary

The *Schistosoma mansoni* Venom Allergen Like proteins (SmVALs) have been identified in the Transcriptome and Post-Genomic studies as targets for immune interventions. Two secreted members of the family were obtained as recombinant proteins in the native conformation. Antibodies produced against them showed that SmVAL4 was present mostly in cercarial secretions and SmVAL26 in egg secretions and that only the native SmVAL4 contained carbohydrate moieties. Due to concerns with potential allergic characteristics of this class of molecules, we have explored the mouse model of airway inflammation in order to investigate these properties in a more confined system. Sensitization and challenge with rSmVAL4, but not rSmVAL26, induced extensive migration of cells to the lungs, mostly eosinophils and macrophages; moreover, immunological parameters were also characteristic of an allergic inflammatory response. Our results showed that the allergic potential of this class of proteins can be variable and that the vaccine candidates should be characterized; the mouse model of airway inflammation can be useful to evaluate these properties.

## Introduction

Schistosomiasis is an important parasitic disease, caused by trematode worms of the genus *Schistosoma*, affecting more than 200 million people worldwide, with a further 650 million individuals living at risk of infection, remaining a major public health problem in many developing countries [1]. Transmission occurs through human contact with water containing the cercariae, the infective larval stage. These penetrate the skin, maturing into schistosomula, which reach the lungs via the systemic circulation. In the lungs, the young parasites undergo morphological transformations, gathering in the portal system, where they mature into adult worms. After pairing, the onset of egg deposition in the intestinal lumen, leads to a range of morbidities, such as granulomatous inflammation and periportal fibrosis [2]. A fraction of the eggs is eliminated with excreta, reaching the fresh water supply, where the miracidia hatch, infecting *Biomphalaria* snails. From these intermediate hosts the cercariae are released into the water to infect the definitive human host, closing the cycle [3].

Within the publication of the transcriptome data for *Schistosoma mansoni*, a series of novel genes/proteins were selected as potential vaccine candidates based on their functional classification by Gene Ontology functions, which would indicate their surface exposure to allow interaction with the host immune system [4]. Among them, four members of a family of wasp venom allergen orthologs were identified, raising the question of what benefits would there be to the parasite in amplifying allergic or other inflammatory responses in the host interface. Recently, this gene family was formally named as *Schistosoma mansoni* Venom Allergen-Like proteins (SmVALs) and its individual members analyzed concerning the phylogenetic relationships, genomic organization and mRNA expression profile across the life cycle [5]. This work revealed that it is a large family of genes composed by 28 members, with at least 24 members transcriptionally active, which can be divided into two groups; those with a signal peptide that may be released and interact with their immediate environment (group 1), and those without a secretion signal that should play an intracellular role (group 2) [5]. Since it was the first article to deal specifically with this schistosome gene family, herein we will follow their proposed numbering and nomenclature.

Following the transcriptome work, a series of proteomic studies describing different aspects of schistosome life cycle and biology were reported [6], [7], [8], [9], [10], [11], [12], [13], [14], mostly using the sequence database of the *S. mansoni* and *S. japonicum* transcriptomes [4], [15], as well as the recently published genome databases [16], [17]. Noteworthy, were the studies on the released proteins (RP) into the skin during the transition from cercariae to schistosomula [7], [10], since these proteins could be the first ones to be accessible to the immune system. In one of these studies, three different members of the previously described wasp venom allergen orthologs family (SmVAL4, 10 and 18) were identified as potential immunomodulators [7] and, more recently, SmVAL10 and 18 were characterized as glycosylated secreted proteins after cercarial transformation [9]. Moreover, in a report using a more accurate model to mimic cercariae penetrating human skin, SmVAL4 was detected in the forming tunnels as a secreted protein, 2 hours post cercariae invasion [8]. In a study integrating the transcriptome and proteomic data from *S. japonicum*, several orthologs of this protein family were identified [11]; worthy of notice, was an SjVAL ortholog detected in the tegument of schistosomula, sharing 73% of identity with SmVAL26. Also of interest, SmVAL6, a group 2 family member, was identified as a tegument-exclusive protein in a sub-proteome analysis of *S. mansoni* [13] (Figure S1).

In schistosomiasis, morbidity and mortality have been associated with egg deposition, therefore identifying the components of Egg Secreted Proteins (ESP) is important to understand how these antigens can regulate the surrounding cytokine environment. Using the proteomics approach, four different members of the SmVAL family (SmVALs 2, 3, 5 and 9) were identified as ESP [6]. However, in marked contrast, more recently, no one of these proteins were identified in egg secretions by another proteomic study [12], emphasizing how methodological differences can result in diverse conclusions. An additional proteomic study reinforced the wide-spread distribution of this family along the life cycle, by the identification of several different SmVALs (2, 3/23, 9, 15, 26/28, and 27) released during *in vitro* miracidium-to-sporocyst transformation [14]; most of this data are summarized in Figure S1.

A natural question that emerged from all these studies is the biological function of these genes in the host-parasite interface. Some of these molecules were suggested by us and by other groups as potential vaccine candidates or immunomodulators, due to their functional classification, expression profile and predicted localization [4], [5], [7], [8], [9]. Additionally, SmVALs members present sequence similarity to the hookworm lead vaccine candidate NaASP-2 [18], [19], [20]. From a vaccine perspective, a major concern is the potential allergic effects of these molecules. Herein, we tried to investigate the immunomodulatory properties of some SmVALs by exploring the murine model of airway inflammation [21]. The investigation of localized inflammation in tissues is often difficult because it is hard to isolate the immune response against a particular stimulus. Therefore, the utilization of an inflammatory model in a confined location can be useful to monitor changes in cell population and to identify regulatory mechanisms.

We selected SmVALs from group 1, which would be putatively expressed in intra-host stages preferably exposed to interaction with the immune system. Therefore, we selected SmVAL4, which would be released in the transition between cercariae and schistosomula [7], [8] and SmVAL26, which would probably be in the tegument of schistosomula due to its *S. japonicum* ortholog identification in the tegument [11]. The respective recombinant proteins were obtained in an eukaryotic expression system and the purified proteins used to produce specific antibodies. The protein expression profile was characterized across the life cycle stages. The allergic properties of SmVAL4 and SmVAL26 proteins were investigated in the murine model of airway inflammation. Our results show that the allergic properties of these molecules vary significantly and this should be considered in the putative design of a schistosomiasis vaccine.

## Materials and Methods

### Parasite maintenance

*Schistosoma mansoni* adult worms (BH strain) were obtained by perfusion of hamsters, 6 weeks after infection with 200 cercariae; eggs were extracted from infected hamster liver by maceration and partial digestion with collagenase followed by washes and passage through sieves and percoll gradients as previously described [22]; miracidia were obtained by exposing purified eggs to a bright light; cercariae were harvested from infected *B. glabrata* snails exposed to light. Following *in vitro* transformation of cercariae, schistosomula were cultured for 0–6 hours or 7 days prior to recovery [23].

### Ethics statement

The procedures involving animals were carried out in accordance with the Brazilian legislation (11790/2008). All animals were handled in strict accordance with good animal practice and protocols were previously approved by the Ethical Committee for Animal Research of Butantan Institute, under the license number 604/09.

### DNA constructs

#### Genes redesigned, optimized and synthesized

The published DNA sequence of SmVAL4 and SmVAL26 encoding the predicted proteins [5], were redesigned excluding the signal peptide sequences and manufactured by DNA 2.0, Inc. USA (https://www.dna20.com/) using DNA2.0 optimization algorithms for expression in *Pichia pastoris* (Table S1). The fragments corresponding to the mature protein sequences for SmVAL4 (from K22 to E181) and SmVAL26 (from K25 to K176) were digested with *Eco*RI and *Xba*I to generate inserts with overhang ends that were purified and cloned into the same sites for the expression vector pPICZαA (Invitrogen), to produce a protein that contained a C-terminal hexa-Histidine tag. The resulting constructs were sequenced to confirm their identity.

### Post-translational modification prediction

The signal peptide prediction was performed using the SignalP 3.0 server (http://www.cbs.dtu.dk/services/SignalP/), N-glycosylation sites were analyzed using the NetNGlyc 1.0 (www.cbs.dtu.dk/services/NetNGlyc/), O-glycosylation sites were analyzed using the OGPET (http://ogpet.utep.edu/OGPET/), and transmembrane helices were analyzed by TMHMM version 2.0 (http://www.cbs.dtu.dk/services/TMHMM-2.0/). Molecular weight (MW) and isoelectric point (pI) were calculated with the Compute pI/Mw tool (http://www.expasy.ch/tools/pi_tool.html).

### Expression of rSmVAL4 and rSmVAL26 in *Pichia pastoris*

The plasmids containing SmVAL4 (optimized sequence) and SmVAL26 (optimized sequence) were linearized with *Sac*I and the *P. pastoris* strain GS115 (Invitrogen) was transformed by electroporation following the instructions of the manufacturer. Twenty colonies were first isolated and purified in YPDS plates containing 100 µg/mL Zeocin, to select putative multi-copy recombinants in YPDS plates containing 500, 1000, and 2000 µg/mL Zeocin. To verify production of the relevant proteins, initial studies were done in small-scale expression conditions, followed by Western blot with anti-His-tag antibody (GE).

Fermentation conditions were carried out as per manufacturer's recommendations. Briefly, selected *P. pastoris* cells were grown in 15 mL of BMGY 28–30°C in a shaking incubator (250–300 rpm) until cultures reached an OD_600_ = 2.0 (approximately 16–18 h). The cells were harvested by centrifuging at 3000× g for 5 min at room temperature, the supernatant was decanted and cells resuspended in 20 mL of BMMY medium to induce expression. Methanol was added to a final concentration of 0.5% methanol every 24 h to maintain induction; expression was monitored at 48 and 96 h time points. The supernatants and cell pellets for 15 colonies of each SmVAL was analyzed for protein expression by Western blot. Those colonies that presented the highest expression level were selected for scale-up fermentation.

### Purification of rSmVAL4 and rSmVAL26 from *P. pastoris*

For protein expression and purification, selected clones for SmVAL4 and SmVAL26 were grown (28°C, 250 rpm) in 25 mL of BMGY in a 250 mL baffled flask until OD_600_ = 2.0 (approximately 18 h), then inoculated in 300 mL of BMGY in a 2.0 L baffled flask and grown in the same conditions until culture reached OD_600_ = 2−6. The cells were harvested (3000× g, 5 min at room temperature) and resuspended in 600 mL of BMMY to start induction. Methanol was added to a final concentration of 0.5% every 24 h to maintain the induction. Cells were harvested after 48 h (SmVAL26) and 96 h (SmVAL4) by centrifugation. The culture medium containing the secreted proteins (rSmVAL4 and rSmVAL26) were filtered through a 0.22 µm membrane, and diluted with 3 volumes of equilibration buffer (50 mM sodium phosphate pH 5.8 (for rSmVAL4) and pH 7.2 (for rSmVAL26), 150 mM NaCl, 20 mM imidazole). The recombinant proteins were then purified by metal affinity chromatography using the Akta Prime system (GE Healthcare) under native conditions. Briefly, the sample was loaded onto a Ni^2+^-NTA column (5 mL bed volume) pre-equilibrated with the same buffer. The column was washed with 30 bed volumes of the equilibration buffer and then eluted with 20–500 mM imidazole linear gradient. Fractions encompassing the main peak and the purity of the preparation were assessed by SDS-PAGE. Before its use the proteins were dialyzed in Phosphate Buffer Saline pH 7.4 (PBS).

### Circular dichroism (CD) measurements

CD measurements were carried out on a Jasco J-810 Spectropolarimeter at 20°C equipped with a Peltier unit for temperature control. Far-UV CD spectrum was acquired using a 1 mm path length cell at 0.5 nm intervals over the wavelength range from 190 to 260 nm. Five scans were averaged for each sample and subtracted from the blank average spectra. The protein concentration was kept at 10 µM in 10 mM sodium phosphate buffer pH 7.4.

### Protein expression profile and N and O-deglycosylation assays

Total protein extracts from whole parasite stages (eggs, miracidia, cercariae, *in vitro* 7-day-old schistosomula and adult worms) were prepared in 40 mM Tris, pH 7.4, 2% SDS, plus protease inhibitor cocktail (Sigma) by sonication (4 cycles of 2 min, with pulses of 0.75 s, 40% amplitude). The samples were centrifuged at 20,000× g for 30 min at 4°C and the supernatant was recovered and used for the assays. The tegument extract was obtained by a freeze/thaw/vortex procedure, as previously described [24]. Their protein concentrations were determined with a DC Protein Assay Kit (Bio-Rad, CA, USA). Purified rSmVALs (50 ng), total parasite protein extracts (20 µg), and total tegument extract (20 µg) were subjected to SDS-PAGE. The gel was electroblotted onto a PVDF membrane, which was blocked with 0.02 M Tris (pH 7.5) and 0.3% Tween 20 containing 5% dry milk for 16 h at 4°C. Subsequently, the membrane was incubated in 1∶4000 or 1∶3000 dilution of anti-rSmVAL4 and anti-rSmVAL26 primary antibody, respectively, in blocking buffer plus 150 mM NaCl for 3 h at room temperature. After three washes using 150 mL of 10 mM Tris (pH 7.5), the membrane was incubated in a 1∶2000 dilution with secondary goat anti-mouse IgG conjugated to horseradish peroxidase (Sigma) for 1 h, followed by another three washes using the same buffer. Antibody reactivity was developed with ECL reagent (GE Healthcare) according to the manufacturer's instructions and imaged using Hyperfilm (GE Healthcare).

N-deglycosylation of native and recombinant proteins was carried out as previously described [25]. Briefly, 20 µg of parasite extracts (in 40 mM Tris, pH 7.4, 0.7% SDS, 1% 2-mercaptoethanol) or 10 µg of recombinant proteins (in PBS, pH 7.2, 0.7% SDS, 1% 2-mercaptoethanol) were denatured by boiling for 10 min. NP-40 (Sigma) was added (1% final concentration), 2 µL of recombinant N-glycosidase F (500 U/µL) (New England Biolabs), 20 mM sodium phosphate (pH 7.5) for a final volume of 20 µL, and incubated overnight at 37°C. For subsequent O-deglycosylation of native proteins the following enzymes were used α(2→3,6,8,9) Neuraminidase, O-glycosidase, β(1→4)-Galactosidase and β-N-Acetylglucosaminidase, and the reactions were carried out as per the manufacturer's recommendations (Sigma). Samples of purified rSmVALs and protein parasite extracts digested and non-digested were submitted to SDS-PAGE. The gels containing the recombinant proteins before and after treatment with glycosidases were stained with Schiff's reagent (Sigma) for detection of glycoproteins as per the manufacturer's recommendations; as control for the specificity of the reaction, we used Bovine Serum Albumin (Bio-Rad) at the same concentration of the recombinant proteins. To analyze the glycosylation pattern of native SmVAL proteins, the protein parasite extracts treated with glycosidases were electroblotted onto a PVDF membrane and Western blot developed as described above.

### Processing cercariae released proteins

Shedding of cercariae from snails was stimulated by exposure to bright light. Mechanical transformation by vortexing was used to stimulate the release of gland cell contents of 2000 parasites during a time course of 0–6 h culture period in 2 mL of RPMI 1640 medium (Invitrogen) containing 300 units/mL penicillin and 300 g/mL streptomycin at 37°C in 5% CO_2_. Parasites were collected by centrifugation at 200× g at 4°C for 5 min and processed as previously described; the supernatant (medium containing released proteins) was stored at −20°C with the addition of 2 µL of 10× general use protease inhibitor mixture (Sigma). This soluble preparation, termed the 0–6 h released proteins (RP), was precipitated with trichloroacetic acid (TCA) and used for Western blot analysis.

### Collection of egg secreted proteins (ESP) and the hatched fluid (Hf)

For egg released protein collection, eggs were isolated as previously described [22], followed by a separation of mature and immature eggs as previously described [26]. ESP was collected by incubating 3.0 million mature eggs in 10 mL of serum-free RPMI (Invitrogen) for 72 h at 37°C in 5% CO_2_. Post-culture viability of eggs was >85%, as assessed by observation of muscular and flame cell activity in unhatched miracidia. Culture medium containing ESP was precipitated with TCA and resuspended in 40 mM Tris, pH 7.4, 2% SDS, plus protease inhibitor cocktail (Sigma) for Western blot analyses. The Hf was obtained by hatching mature eggs through exposure to bright light for 1 h in pound water. Miracidium and egg shell were pelleted by centrifugation at 200× g at 4°C for 10 min, and the protein water-soluble content was carefully collected to avoid turbulence, the sample (Hf) was filtered through a 0.22 µm polyethersulfone filter (Millipore) and concentrated through precipitation with TCA.

### Pronase protein digestion

For elimination of the protein moiety, 12 µg of rSmVAL4 was incubated with 0.05% of pronase (Calbiochem, San Diego, CA) for 2 h at 37°C. A separate sample of rSmVAL4 was incubated for 2 h at 37°C without pronase to provide an undigested control. Successful pronase digestion was confirmed by gel electrophoresis followed by Coomassie staining, which revealed the absence of any remaining protein (data not shown).

### rSmVALs sensitization and challenge

Female BALB/c mice (6–8 wk old) were weight matched and used throughout this study. To test whether rSmVALs could induce an inflammatory reaction in the lungs, we adapted the well-established murine model of asthma based on Ovalbumin (OVA)/alum sensitizations and challenges [21]. Briefly, mice were sensitized on days 0, 7 and 14 by subcutaneous (s.c.) injection (0.4 mL total volume) in the nape of the neck with 10 µg rSmVALs adsorbed to 2.0 mg of aluminum hydroxide (Alhydrogel-Brenntag Biosector). On days 21 and 28, mice were challenged i.n. with 10 µg of rSmVALs in 50 µL of PBS. Two additional groups were used: Control group received only the intranasal challenge with rSmVAL and naïve mice received only PBS. It is important to mention that mice were anesthetized intramuscularly with 100 µL of a solution containing ketamine (Ketamina Agener, Uniao Quimica Farmaceutica Nacional) and xylazine (Bayer) before any immunization or challenge, to ensure a complete instillation to the lungs, as previously described [27].

### Bronchoalveolar lavage (BAL)

Twenty four hours after the last challenge, mice were deeply anesthetized by an i.p. injection of urethane (Sigma-Aldrich) at 15 mg/10 g body weight, the abdominal cavity was opened, and blood samples from the inferior cava vein were collected for serum antibody determinations. The trachea was cannulated and lungs were lavaged twice with 0.5 and 1.0 mL of cold PBS. After total cell counting, cytospin preparations of bronchoalveolar lavage (BAL) cells were stained with Instant-Prov (Newprov) and differential cell counts were performed on 200 cells on the basis of morphology and staining characteristics. Supernatants from BAL were collected and frozen at −80°C for cytokines measurements.

### Measurement of rSmVALs-specific IgG1, IgG2a and IgE antibodies

Anti-SmVALs antibodies were assayed by sandwich ELISA, as previously described [21]. Briefly, serum samples were titrated for optimal dilutions for testing different isotypes. For SmVALs-specific IgE determinations, plates were coated with goat anti-mouse unlabelled IgE (1∶250; BD Bioscience) following the manufacturer's recommendations; serum samples were incubated (1/10 dilution) for 2 h at room temperature and subsequently biotin-labeled rSmVALs (5 µg/mL) were added to the wells. The biotinylated rSmVALs were prepared by reacting 1 mL rSmVALs (1 mg/mL) in PBS with 100 µL of N-hydroxysuccinimidobiotin in dimethyl sulfoxide (DMSO) (4 mg/mL) for 4 h at room temperature, followed by overnight dialysis against PBS at 4°C. The bound rSmVALs-biotin was coupled to streptavidin-peroxidase 1∶250, for 15 min incubation at room temperature and revealed as per the manufacturer's recommendations. SmVAL-specific IgE levels of samples were expressed by OD. For SmVALs-specific IgG1 and IgG2a antibodies, serum samples were plated on 96-well plates previously coated with rSmVALs (0.5 µg/well). The bound antibodies were revealed with goat anti-mouse IgG1 or IgG2a followed by peroxidase-labeled rabbit anti-goat antibodies (all from Southern Biotech). The concentration of each specific isotype was estimated by comparison with IgG1 and IgG2a standards run in parallel and expressed as the mean ± SEM of the antibody concentration of 4 mice per group.

### Cytokine measurements

The cytokine concentration in the BAL fluid was quantified by ELISA kits specific for IL-5 and IL-10 (BD Biosciences PharMingen) and for IFN-γ (Peprotech INC.). The values are expressed as picograms per milliliter deduced from standards, run in parallel with the recombinant cytokines. The limit of detection values were 10 pg/mL for IL-5 and IL-10 and 16 pg/mL for IFN-γ.

### Passive cutaneous anaphylaxis (PCA)

The anaphylactic activity of reactogenic antibodies was evaluated by passive cutaneous anaphylactic reaction in mice as described by Ovary et al. [28]. Previously shaved mice were injected intradermally with 50 µL of three serial dilutions of serum in each side of the dorsal skin. After 2 h, they were challenged intravenously with 250 µg of rSmVAL4, rSmVAL4-Pro (Pronase-digested) or rSmVAL26, all plus 0.25% of Evans blue solution. All determinations were made in triplicate and the PCA titers were expressed as the reciprocal of the highest dilution that gave a lesion of >5 mm in diameter. The detection threshold of the technique was established at 1/5 dilutions.

### Immunization of mice and polyclonal antibody production

Polyclonal mouse sera were produced against preparations of rSmVAL4 and rSmVAL26. BALB/c mice were immunized three times, subcutaneously in the nape of the neck, at 14-day intervals with 25 µg of rSmVAL4 or rSmVAL26 formulated with TiterMax adjuvant (CytRx Corporation; first dose) or PBS 1x (in subsequent doses). Fifteen days after the last inoculation, rodents were exsanguinated. The sera were used at a dilution of 1∶4000 (anti-rSmVAL4) and 1∶3000 (anti-rSmVAL26) in Western blots.

### Statistical analysis

Student's *t*-test was used to compare experimental and control groups on antibody and cytokine levels. For cellular migration assays and analysis involving more than two groups, statistical comparisons were performed with one-way ANOVA followed by a Bonferroni pairwise comparison. A ρ value<0.05 was considered statistically significant.

## Results

### Construction, expression and purification of recombinant SmVAL4 and SmVAL26

In order to analyze SmVALs that could interact with cells in the definitive host, we chose 2 members of group 1. SmVAL4 would be released in the transition between cercaria and schistosomula. A phylogenetic analysis of SmVALs and SjVALs (Figure S2), revealed that SmVAL26 branched together with a SjVAL ortholog, with 73% of identity detected in the tegument of hepatic schistosomula (Gene Bank accession AAW27353.1)(Figure S3).

**Figure 1:**
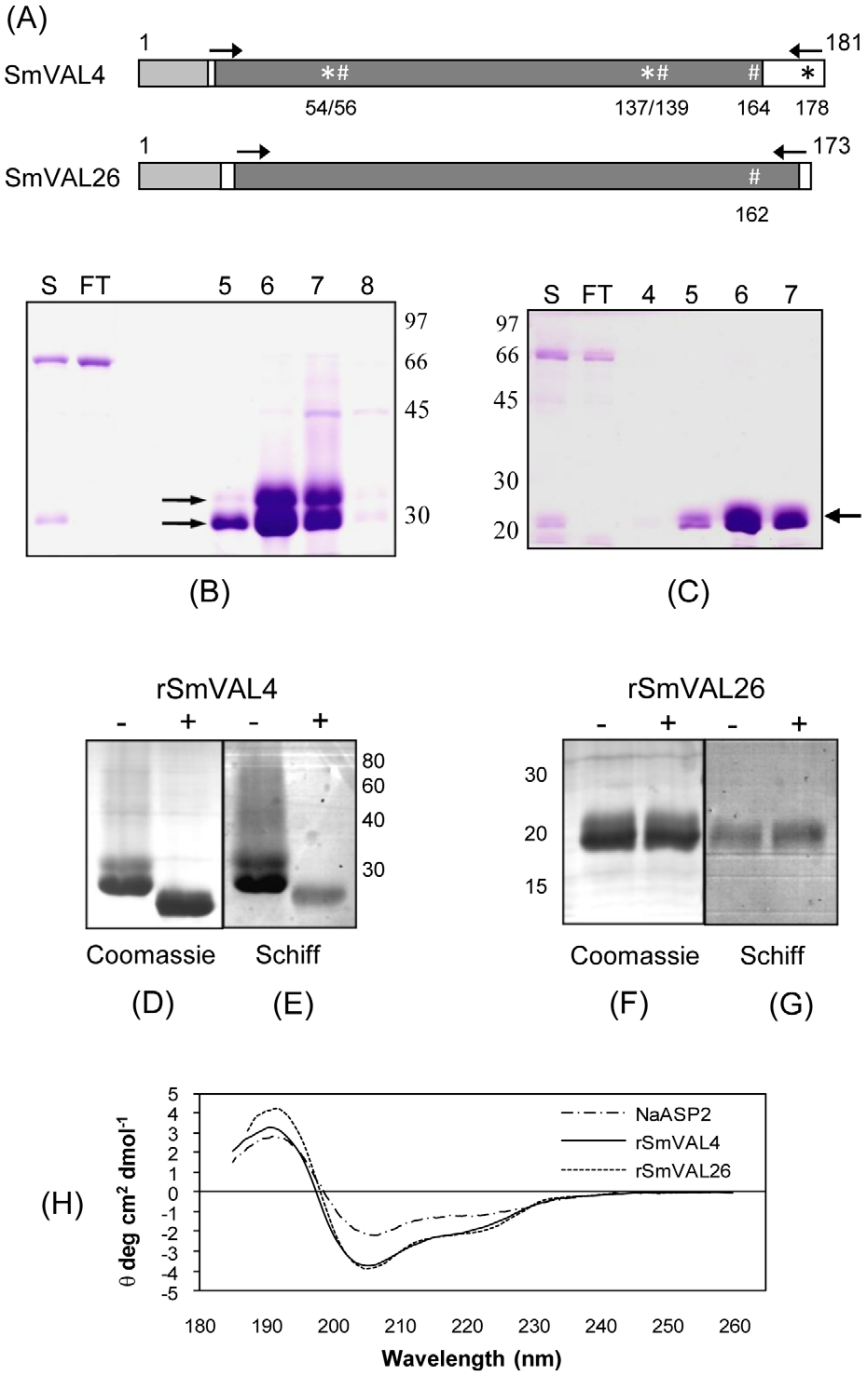
Expression and purification of rSmVAL4 and rSmVAL26, and characterization of their glycosylations. (A) Schematic representation of SmVAL proteins used in this study. Highlighted are the putative signal peptide (grey box), the SCP signature sequence (dark grey box), potential sites for N-glycosylation (*) and O-glycosylation (#), and the cloned region expressed in *Pichia pastoris* (<$>\scale 65%\raster="rg1"<$>). Numbers correspond to the amino acid sequence position deduced from the cDNA sequence. (B) SDS-PAGE analysis of fractions from Ni^2+^-charged column chromatography of rSmVAL4 and (C) rSmVAL26; Lanes 5–8 and 4–7, fractions containing the main peak of rSmVAL4 and 26 respectively, eluted by linear gradient of imidazole (20–500 mM); FT - Flow-through; S – sample. (D) SDS-PAGE of rSmVAL4 and (F) rSmVAL26 stained with Coomassie before treatment with PNGase F (−) and after digestion with the enzyme (+); (E and G) the same gel was stained with Schiff's reagent. Positions of molecular mass standards (kDa) are indicated on the side, 20 µg of protein was loaded in each lane. (H) Circular Dichroism spectra of rSmVAL4 and rSmVAL26 are compared to that of the rNaASP-2 spectra. The CD spectra presented are the averages of five measurements.

SmVALs contain the sperm-coating protein (SCP) signature sequence (outlined by a dark grey box in Figure 1A) and are recognized as part of the Pfam SCP family (PF00188) with an E-value ranging from 6.66×10^−24^ to 8.20×10^−31^. The SmVALs under investigation, once belonging to group 1, also contain a putative N-terminal signal peptide (outlined by a grey box in Figure 1A). Putative N and O-glycosylation sites were identified and investigated (identified by * and #, respectively in Figure 1A). The predicted molecular mass and isoelectric points of these proteins are presented in Table 1.

**Table 1:** Molecular weight and isoeletric point of SmVALs investigated in this study.

	SmVAL	aExp Mw	bObs Mw	pI
**Native**	SmVAL4	18.5	29.0	8.68
	SmVAL26	17.3	19.0	5.26
**Recombinant**	SmVAL4	21.4	30.0	7.21
	SmVAL26	20.2	20.0	5.35

a Exp Mw = Expected Molecular weight.

b Obs Mw = Approximate Observed Molecular weight.

### rSmVAL4 and rSmVAL26

The recombinant proteins rSmVAL4 and rSmVAL26 were expressed using codon optimization in *P. pastoris* GS115 strain and secreted into the culture supernatant (products around 30 kDa and 20 kDa, respectively) (Figure 1B and 1C). rSmVAL4 and rSmVAL26 were purified by affinity chromatography on nickel-charged columns eliminating the main contaminant from both samples (around 66 kDa) in the flow through (Figure 1B and 1C). The eluted fractions of rSmVAL4 showed two main bands at ∼30 and 34 kDa (Figure 1B lanes 5–8), while rSmVAL26 eluted fractions presents only one product (∼20 kDa) (Figure 1C, lanes 4–7). Eluted fractions were pooled and submitted to extensive dialysis in PBS pH 7.2; protein yield after dialysis were estimated to be around 10.0 mg of rSmVAL4/L and 6.0 mg of rSmVAL26/L of culture. These samples were used in the sensitization and challenge assays and to generate polyclonal antibodies in mice.

### rSmVAL4 and rSmVAL 26 are expressed as glycosylated proteins

Both *Pichia*-secreted proteins migrate with a higher molecular mass than that predicted (∼30.0 kDa for rSmVAL4 and ∼20 kDa for rSmVAL26) (Table 1), which could reflect a likely product of post-translational glycosylation. To test this hypothesis, we digested the purified proteins with a recombinant N-glycosidase F, and also stained the gels with Schiff's reagent to reveal the presence of glycans. The SDS-PAGE showed that, after digestion, rSmVAL4 migrated to a lower MW (∼25 kDa), demonstrating the removal of N-glycans (Figure 1D), while rSmVAL26 does not show any mobility shift (Figure 1F), suggesting that the protein was not N-glycosylated. Additionally, for both proteins, the Schiff's reagent staining procedure revealed the presence of glycans after treatment with PNGase F, suggesting the presence of a PNGase F insensitive N-linked glycan site or an O-linked glycosylation site (Figure 1E and G).

### rSmVAL4 and rSmVAL26 present an ordered secondary structure

Circular dichroism spectra revealed that rSmVAL4 and rSmVAL26 display an ordered secondary structure, which resembles the NaASP-2 protein (structure resolved – three layer α-β-α-sandwich) (Figure 1H), although the proportions of secondary structure elements (α-helix and β-sheet) were not calculated.

### SmVALs protein expression profile across the parasite life cycle

**Figure 2:**
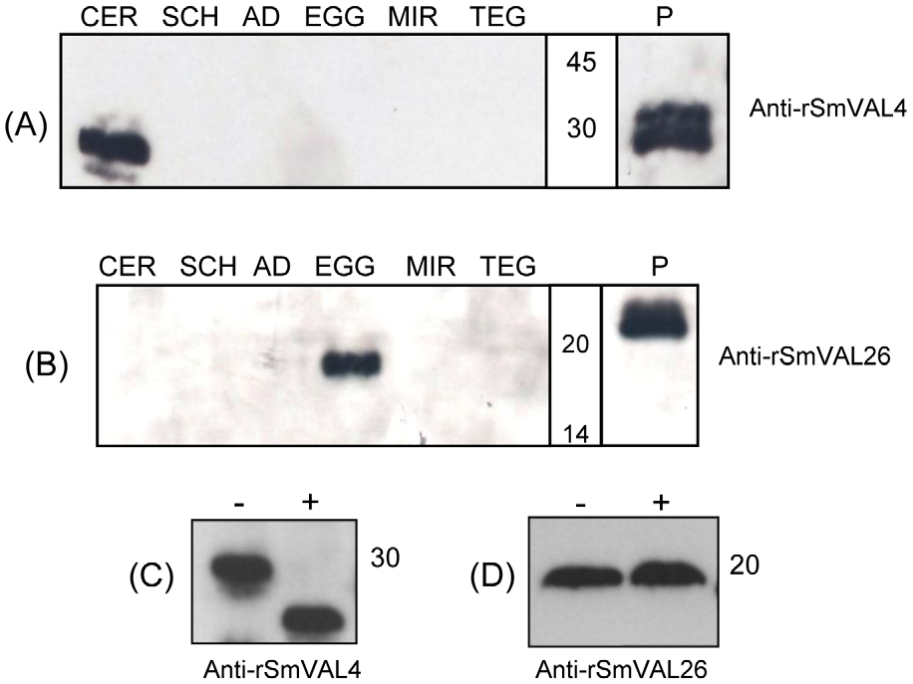
Immunoblotting of protein extracts from *S. mansoni* stages and tegument fraction using anti-rSmVALs polyclonal antibodies. (A) Anti-rSmVAL4, and (B) anti-rSmVAL26. CER, cercariae; EGG, eggs; MIR, miracidia; AD, adult worms (female and male); SCH, 7-day old schistosomula; TEG, tegument; (C) Immunoblotting of Cercariae extracts before (−) and after (+) treatment with PNGase F using anti-rSmVAL4; and (D) Immunoblot of Egg extracts before (−) and after (+) treatment with PNGase F using anti-rSmVAL26 (20 µg of protein was loaded in each lane); P, positive control rSmVALs (50 ng). Positions of molecular mass standards (kDa) are indicated inside or on the side the autoradiogram film.

Samples prepared from cercariae, schistosomula, adult worms, eggs and miracidia stages of *S. mansoni*, and tegument isolated by the freeze/thaw method, were all separated by SDS-PAGE. Immunoblotting was performed using mouse anti-rSmVAL4 and rSmVAL26 antisera. The protein expression profile of SmVALs revealed a very specific stage associated expression. Briefly, the expression of SmVAL4 seems to be restricted to the cercariae stage. In the case of SmVAL26, although we expected it to be in the tegument due to its similarity with the *S. japonicum* ortholog, it is actually detected in the egg, but not in the miracidium stage (Figure 2A and B). It is important to note that in the assessed experimental conditions, no sign of cross reactivity was observed with SmVALs in other stages or in the tegument fraction.

### Native SmVAL4 is an N-glycosylated protein

Noteworthy, the SmVAL4 and SmVAL26 native proteins detected in schistosome extracts migrate with a higher molecular mass than that predicted (Table 1), which again could reflect a likely product of post-translational glycosylation. To test this hypothesis, we digested total cercariae and egg extracts with the recombinant N-glycosidase F. The immunoblot showed that, after digestion, the native proteins displayed a shift in the migration, demonstrating that native SmVAL4 is N-glycosylated, whereas SmVAL26 is not (Figure 2C and D). In order to investigate possible O-glycosylations, the extracts were treated with neuraminidase, O-glycosidase, beta (1–4) galactosidase, or N-acetylglucosaminidase, all of which had no effect on the proteins' mobility on SDS-PAGE (data not shown).

### SmVAL4 is released by cercariae and SmVAL26 is released by eggs

**Figure 3:**
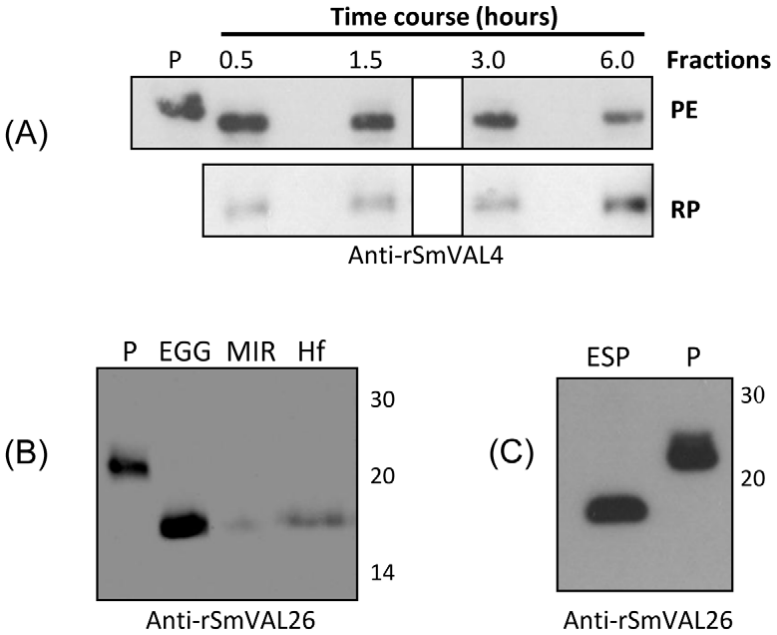
Immunoblotting of released proteins using anti-rSmVALs polyclonal antibodies. (A) Released proteins by newly transformed schistosomulum (RP) cultured 0–6 h or correspondent parasite extract (PE) hybridized with anti-rSmVAL4 (RP - total released proteins by 1000 parasites was loaded in each lane, PE - 10 µg of protein extract was loaded in each lane). (B) EGG, eggs; MIR, miracidia; Hf, hatched fluid containing released proteins by hatching eggs, hybridized with anti-rSmVAL26; (C) ESP, secreted proteins by 72 h cultured viable mature eggs, hybridized with anti-rSmVAL26, (20 µg of protein was loaded in each lane); P, positive control rSmVALs (50 ng).

To investigate the presence of SmVAL4 in the secretions of newly transformed schistosomula, we collected the proteins released by 2000 parasites in a time course manner. We were able to detect the secretion of SmVAL4 as early as 30 min after transformation and its increasing secretion in the medium of cultured schistosomula from 0–6 h (Figure 3A). However, after this period, there are still significant amounts of protein within the parasite or associated to its surface (Figure 3A).

We also evaluated the presence of SmVAL26 in the egg secretions and in the hatched fluid, using the respective antibody. SmVAL26 was detected in both extracts (Figure 3B and C).

### rSmVAL4 is able to induce airway inflammatory responses

**Figure 4:**
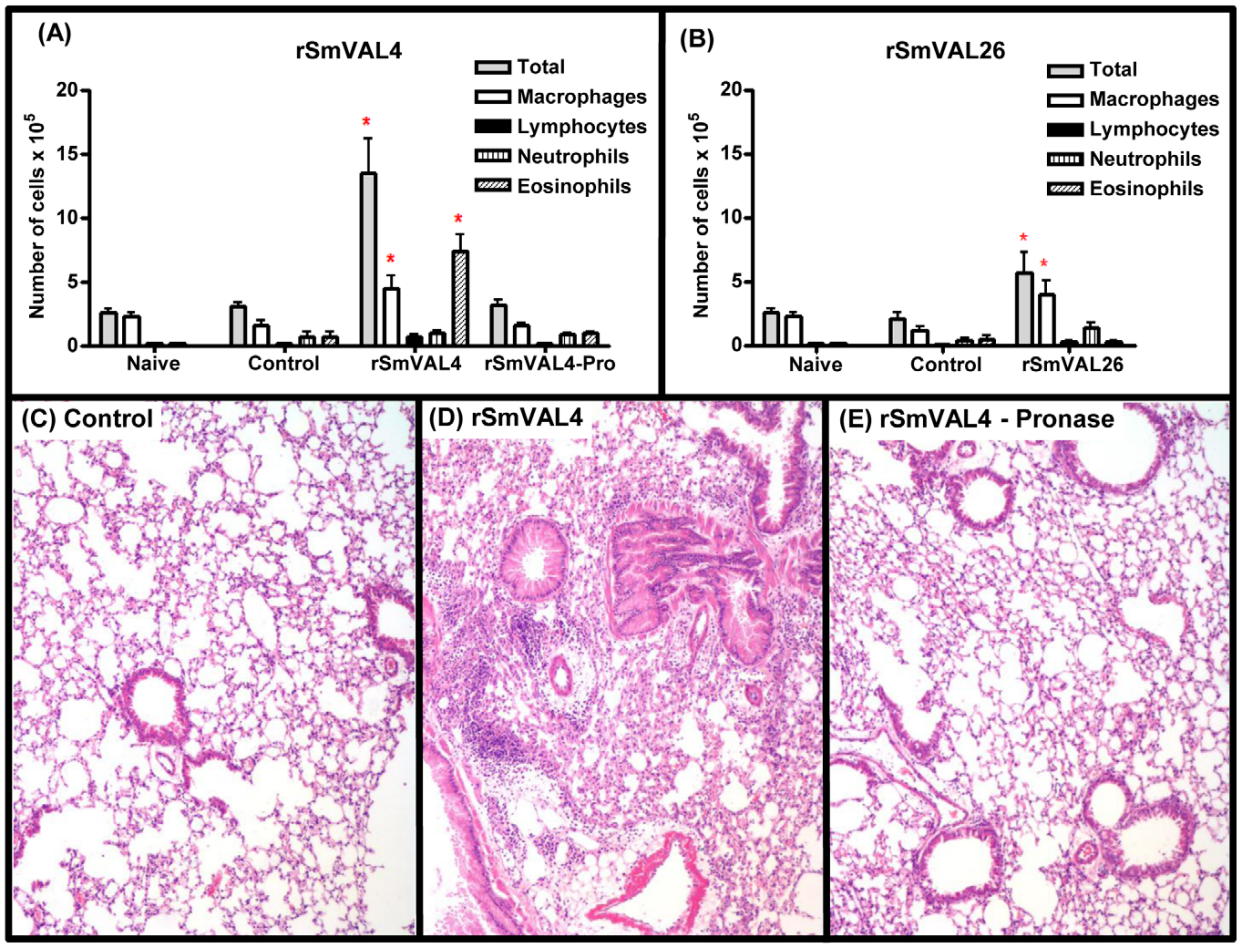
Evaluation of rSmVALs induction of airway inflammation and lung histopathology. (A) BAL total and differential cell counts for animals immunized with rSmVAL4 or rSmVAL4-Pro (rSmVAL4 protein after treatment with Pronase) and (B) rSmVAL26. Control group received only the intranasal challenge with rSmVAL and naïve mice received only PBS. Results are expressed as means ± SEM for groups of four mice and are representative of two experiments. *Significant differences (p<0.05) when compared to Control (mice that were only challenged with the respective proteins). (C) Lung sections of mice that were only challenged. (D) Representative lung sections of rSmVAL4-induced allergic airway inflammation in BALB/c mice, revealing the marked infiltration of inflammatory cells in the peribronchiolar space. (E) Lung sections of mice that received rSmVAL4-Pro, showing patterns similar to the control group; pictures at 10× of magnification.

In order to investigate a putative immunomodulatory effect of rSmVALs, we explored the well-established murine model of airway inflammation induced by OVA/Alum sensitization and OVA intranasal challenge, replacing OVA by the rSmVAL4 and rSmVAL26 proteins. Our data revealed that mice sensitized and challenged with rSmVAL4 present an increased number of total cells in the bronchoalveolar lavage (BAL), when compared with the control group. This effect comprises mainly an increase in eosinophils (55%) and macrophages (32%), which resembles an allergic airway inflammatory response. Mice that received rSmVAL26 show a discrete increase in total cell counting, mostly macrophages (Figure 4A and B). When mice received up to 6 doses of rSmVAL26, this profile does not change significantly. The inflammatory response induced by rSmVALs was also evaluated in the lungs of mice by histopathology. A dense mixed-cellular infiltrate surrounding the airway (peribronchovascular inflammation) was evident only in the group treated with rSmVAL4 (Figure 4D).

Since the allergic effect could be due either to the proteic or the carbohydrate moieties of rSmVAL4 produced in *Pichia pastoris*, we eliminated the protein moiety by pronase treatment and performed the sensitization step with the pronase-treated rSmVAL4 (rSmVAL4-Pro). As shown in Figure 4A and 4E, pronase treatment of rSmVAL4 totally abolished airway inflammation.

### rSmVALs induce specific IgG1, but only rSmVAL4 induces IgE production

**Figure 5:**
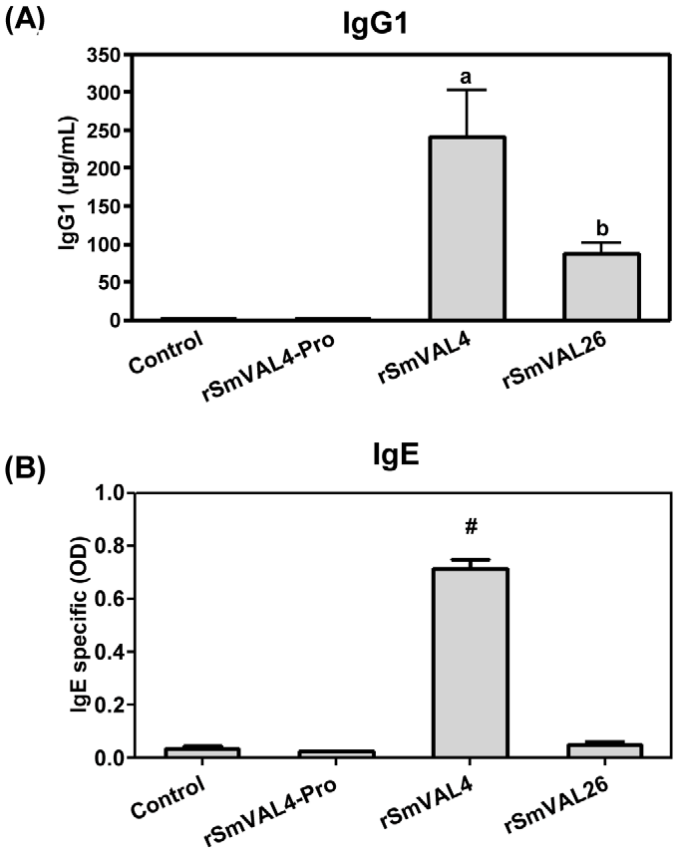
rSmVALs specific antibody production. BALB/c were sensitized s.c. with rSmVALs/Alum, rSmVAL4-Pro/Alum or PBS/Alum (Control) and then challenged i.n. with the proteins. The experiments were performed 24 h after the last challenge. (A) rSmVALs-specific IgG1 and (B) rSmVALs-specific IgE were determined in the sera by sandwich ELISA. Results are expressed as means ± SEM for groups of four mice and are representative of two experiments. a, b or #, significant differences (p<0.05) when compared to Control group (mice that were challenged with protein only).

The serum levels of SmVALs-specific IgG1, IgG2a and IgE were measured in sensitized and challenged mice and in those only challenged with different rSmVAL proteins. The production of SmVAL-specific IgG1, a Th2-affiliated antibody isotype, was significantly higher in all groups analyzed as compared to the control challenge group (Figure 5A). Additionally, rSmVAL4 showed higher levels of IgG1, and no antibody production was observed in response to immunization with pronase-treated rSmVAL4. Concerning the production of SmVALs-specific IgG2a antibodies, very low levels and no significant differences were detected between experimental and control groups (data not shown). The serum levels of SmVALs-specific IgE, a Th2 allergic associated isotype, were also measured, revealing significantly higher levels only in the rSmVAL4 group as compared to all other groups (Figure 5B).

### rSmVAL4 induces high levels of IL-5 but does not induce IFN-γ or IL-10

**Figure 6:**
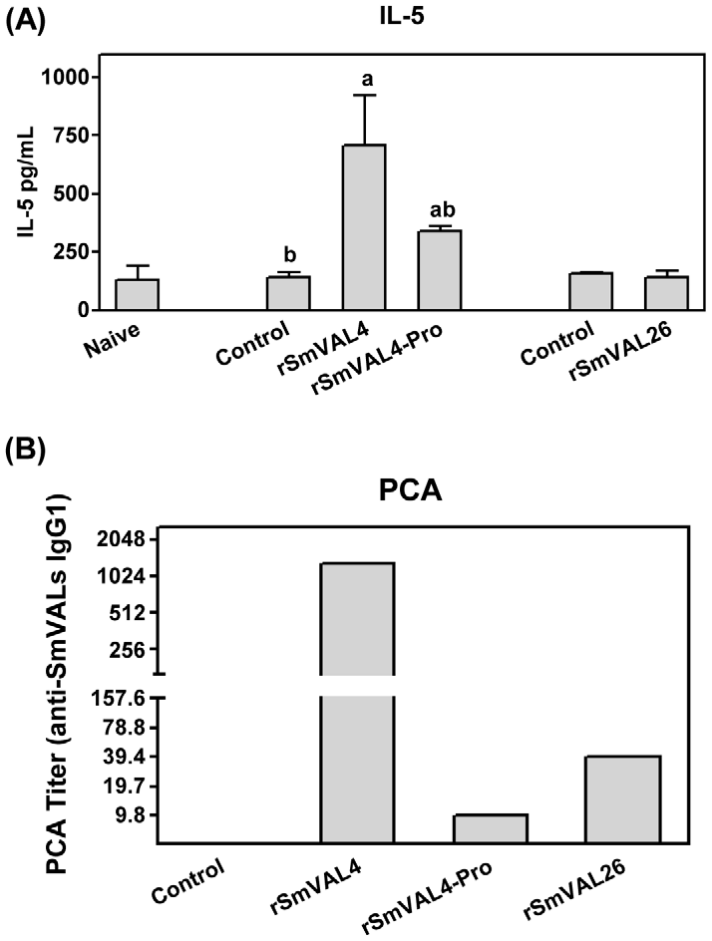
IL-5 and anaphylactic IgG1 detection. (A) IL-5 production in BAL fluid after sensitization and challenge with different rSmVALs. Results are expressed as means ± SEM for groups of four mice and are representative of two experiments. a and b represent statistically significant differences (p<0.05) between groups. (B) Anaphylactic IgG1 antibodies produced after sensitization and challenge with different rSmVALs; PCA titers represent the reciprocal of the highest dilution of heat-inactivated plasma that gave a lesion of 0.5 mm in diameter.

We evaluated the secretion of IL-5, IL-10 and IFN-γ in the BAL fluid. The levels of IFN-γ were below detectable levels (50 pg/mL) in all analyzed groups, while the levels of IL-10 did not differ significantly from the sensitized and challenged groups to those only challenged (data not shown). The levels of IL-5 were significantly higher in the rSmVAL4 immunized mice as compared to the control groups (naïve or only challenged). SmVAL4-Pro group revealed intermediate levels of this cytokine, not differing statistically from control or rSmVAL4 (Figure 6A). It is important to mention that this IL-5 secretion observed in SmVAL4-Pro was not sufficient to induce/mediate the eosinophil infiltration or other parameters of the allergic response.

### rSmVAL4 serum is able to cause passive cutaneous anaphylactic hypersensitivity

Taking into account the eosinophil migration, the presence of IL-5 in the BAL and the high levels of systemic IgE, we performed Passive Cutaneous Anaphylactic (PCA) assays to evaluate the production of anaphylactic IgG1 in heat-inactivated serum of mice sensitized and challenged with rSmVALs. Our results demonstrated that IgG1 antibodies produced by the rSmVAL4 group exhibited strong PCA activity with a very high titer of 1∶1250, whereas in the other groups, including the pronase-treated rSmVAL4 or the control groups, low levels of anaphylactic IgG1 antibodies were observed (Figure 6B).

### rSmVAL4 induces IgE in a conventional protocol of immunization

**Figure 7:**
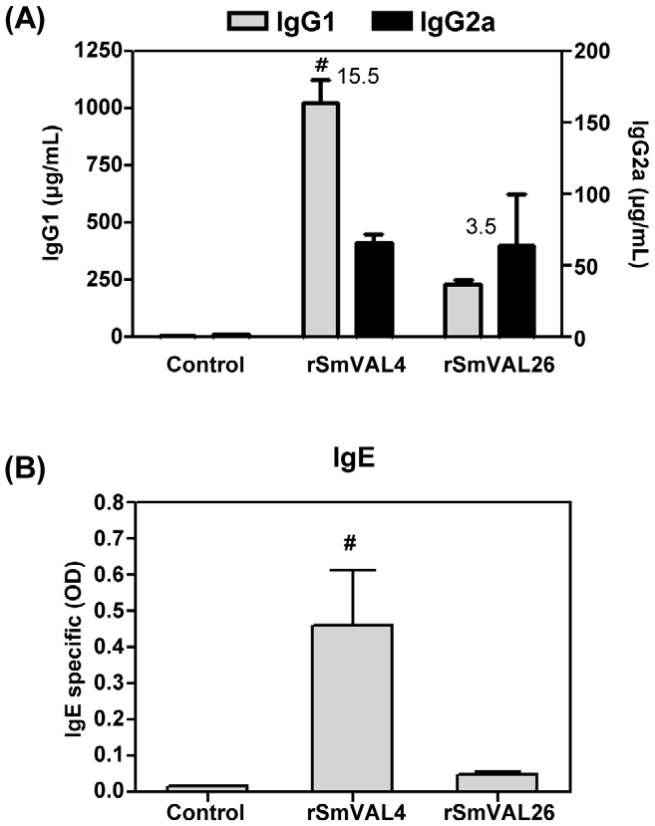
IgG1 and IgG2a immune profile induced by immunization of mice with rSmVALs formulated with TiterMax. BALB/c mice were immunized s.c. with 3 doses of recombinant SmVALs formulated with TiterMax adjuvant (first dose) or PBS 1x (subsequent doses). (A) rSmVALs-specific IgG1 and IgG2a. (B) rSmVALs-specific IgE were determined in the sera by sandwich ELISA. Results are expressed as means ± SEM for groups of four mice. # Significant differences (p<0.05), Control group (pre-bleed serum), ratios between IgG1/IgG2a are presented above the bars.

The induction of high levels of systemic IgE by rSmVAL4 in the model of airway inflammation led us to evaluate the use of a less Th2-prone adjuvant in a conventional protocol of immunization. Therefore, mice were immunized with rSmVALs formulated in TiterMax Gold, which is described to produce considerable levels of IgG2a in addition to IgG1. The rSmVAL4 group showed higher levels of IgG1 antibody in relation to rSmVAL26. Concerning the production of SmVALs-specific IgG2a antibodies, significant levels were detected in both immunized groups as compared to the control, with no differences between them. The levels of specific IgG1 and IgG2a and the IgG1/IgG2a ratio indicate that immunization with TiterMax Gold induced a predominant Th2 immune response to rSmVAL4 and a more balanced (Th1/Th2) response to rSmVAL26 (Figure 7A). The serum levels of SmVALs-specific IgE, were also measured, revealing significantly higher levels only in the rSmVAL4 group as compared to all other groups (Figure 7B).

Animals sensitized with rSmVAL4 and rSmVAL26 formulated in Titermax were also challenged intranasally and airway inflammation evaluated. It was observed that only the group receiving rSmVAL4 developed airway eosinophilic inflammation (data not shown).

## Discussion

Recently, SmVALs have emerged from transcriptoma, microarray and proteomic studies as potential targets for immune intervention. In the present manuscript, we extend the previous molecular characterization performed by Chalmers et al. (2008), focusing on the protein products of SmVAL4 and SmVAL26, which may play different roles in the parasite-host interface.

Our results describe the expression of the codon-optimized versions of SmVAL4 and SmVAL26 in *Pichia pastoris*. The secretion of these proteins in the yeast expression system allowed purification of the proteins in soluble form. Maybe the most relevant feature when producing a recombinant protein for functional assays is its correct folding. Our CD data indicated that the soluble secreted forms of rSmVAL4 and rSmVAL26 contained an ordered secondary structure, with similar proportions of structural elements (α-helix and β-sheet) as determined for NaASP-2, which presents a three-layer (α-β-α) sandwich flanked by an N-terminal loop and a short cystein-rich C terminus [18].

The presence of glycans in such a class of secreted molecules is somewhat expected. Interestingly, both rSmVAL4 and rSmVAL26 were revealed to be glycosylated, which could help the proper folding and stabilization of the protein. It is important to note though, that the profile of glycosylation obtained in the *Pichia* system will not be equivalent to the native pattern in schistosomes. Concerning the native SmVAL4 protein, we confirmed the *in silico* predictions of its N-glycosylation by mobility shift. On the other hand, the native SmVAL26 was not N-glycosylated, which is also in conformity with the N-glycan *in silico* predictions. We did not detect the presence of O-glycans in the native SmVALs analyzed. However, we cannot completely exclude the presence of this class of carbohydrate, since the deglycosidases used were not specific for schistosomes, which could have modifications of the core structure, impairing or blocking enzymatic digestion.

The previously determined mRNA expression profile across the life cycle suggested that SmVAL4 could be involved in the invasion of the definitive host [5]; this stage associated expression was confirmed at the protein level by our Western blot analysis. SmVAL4 protein was revealed to be present only in the cercarial stage, increasing in the secretions of newly transformed schistosomulum in a time course manner. This data could reflect the fact that not all parasites were at the same metabolic stage during transformation or that the protein continues to be released even after 6 hours post-transformation. The identification of SmVAL4 in cercariae secretions by proteomics suggested that it could be localized in the acetabular glands. However, the detection of a significant amount of protein in 6 h schistosomula implies another localization, which is being investigated by imunohistochemistry and whole mount *in situ* hybridization.

Based on its similarity to a *S. japonicum* ortholog, we predicted that SmVAL26 could be in the tegument of young adults or hepatic schistosomula (14 day-old). However, contrary to our expectations, SmVAL26 was identified in the egg stage and in the hatched eggs' fluid. This is in accord with the proteomic study of Mathieson and Wilson (2010), which compared the contents of egg, miracidium, egg secretions and hatch fluid. Interestingly, we also detected SmVAL26 in the secretions of cultured eggs. Based on our data, we hypothesized that SmVAL26 could be localized in the egg envelope, or as proposed by Mathieson and Wilson (2010), in the protective fluid located between the miracidium and the envelope.

The biological function of SmVALs and orthologs belonging to the SCP/TAPS domain super-family remains unclear. The NaASP-2 protein from *N. americanus*, secreted by the infective larvae, was described to induce neutrophil recruitment *in vivo* in the air pouch model of acute inflammation [29]. Two additional hookworm SCP/TAPS proteins were reported to have immunomodulatory activities. *Ancylostoma caninum* hookworm platelet inhibitor (*Ac*-HPI) exhibits an inhibitory effect on platelet aggregation, acting via glycoprotein Ia/IIa [30], and *A. caninum* neutrophil inhibitory factor (*Ac*-NIF) showed ability to inhibit CD11b/18-dependant leukocyte function [31], [32]. Concerning the SCP orthologs from filarial nematodes, the rOv-ASP-1 from *Onchocerca volvulus*, showed striking features ranging from angiogenic activity in mouse corneas, to being proposed as an adjuvant for bystander proteins due to its ability to bind to APCs and trigger Th1 proinflammatory cytokines [33]. It is clear that no common biological function or activity has been associated with this protein family. And it is possible, as proposed by Hewitson et al. [34], that the SCP domain operates as an adaptable protein framework facilitating the evolution of various specialized functions.

In principle, schistosome proteins implicated in the tissue invasion process, are particularly good candidate antigens for the development of vaccines and drugs. One major concern on the use of these and other SmVALs as vaccine candidates is the potential allergic effects of these molecules. Herein, we have explored the murine model of airway inflammation, substituting OVA for the SmVALs, to investigate the putative allergic responses induced by these proteins. Our results demonstrate that following sensitization and challenge with the different proteins, they present varying properties in regards to the recruitment of inflammatory cells to the BAL fluid. SmVAL4 was shown to induce a marked increase in total cells in the BAL fluid, mostly due to an increase in eosinophil and macrophages, which correlated with increases in IgG1, IgE and IL-5, characterizing a typical allergic response, while SmVAL26 showed no alterations in the allergic parameters in the lungs. Furthermore, the use of a Pronase-treated SmVAL4, strongly supports the conclusion that the allergic properties are due to the protein itself and not to the carbohydrate moiety. We also demonstrated that anti-rSmVAL4 sera presented high titers of anaphylactic IgG1 antibody.

Human Schistosoma infections have been associated with the inhibition of allergies. In this context, the murine model of ovalbumin induced airway inflammation has been used to demonstrate that Schistosome infection, egg extracts or some purified antigens modulate negatively the allergic response induced by OVA treatment by a mechanism mediated by T regulatory cells [35], [36]. Intrinsic properties of the antigens must be important for this modulation, since not all molecules modulate equally the allergic response. It is important to note that our model is quite different, since we have investigated the allergenic properties of the antigens per se.

On the other hand, in the course of a *S. mansoni* infection, two phases of immune responses have been recognized. In the first 3–5 weeks, during which the host is exposed to immature parasites, the immune response has been shown to be Th1-predominant. As the parasites mature, mate and begin to produce eggs at weeks 5–6, the immune response alters markedly to a strong Th2 profile [37]. However, there are reasons to believe that responses to schistosome worms during established infections are more complex. For example, immune responses classically mediated by the Th2 cytokine IL-5 (eosinophilia and eosinopoiesis) were reported in the first weeks of infection [38]. Moreover, the induction of CD4^+^ T cells, specific IgE and basophils that produce IL-4 in response to worm antigens (e.g. SmCB1 – Cathepsin B) have also been described in this phase [39]. Finally, there is increasing evidence that excretory/secretory molecules from schistosome larvae can stimulate a mixed T helper response in the skin, with evidence of both Th1 and Th2 skewed responses at the site of infection [40]. Therefore, it is possible that SmVAL4 could be an antigen involved in the initial stage of the infection inducing a Th2-predominant response, whereas SmVAL26 may be more important in the late phase of infection.

In a vaccine context, the immunization of mice with rSmVAL4 and TiterMax Gold (a more balanced adjuvant) produced high levels of IgE in a conventional immunization protocol. It is interesting to note, that when challenged i.n. after this protocol, only rSmVAL4 induced airway inflammation (data not shown). This in itself poses risks, since a vaccination regime that promotes IgE production may well elicit undesirable side-effects such as exacerbation of allergy. Furthermore, in a recent report of a second Phase I trial, adult volunteers did experience allergic responses following immunization with the *Necator* ortholog, NaASP-2 [41]; this data advocates for the presence of IgE epitopes in this class of molecules. Therefore, it would be interesting to evaluate the levels of specific IgE antibodies for SmVAL4 in the sera of individuals resident in a schistosomiasis endemic area. The IgE epitopes for the SmVAL family remain to be determined, but it is tempting to conclude that the SmVAL4 protein contains some IgE epitopes absent on SmVAL26. The functional evaluation of other cercariae secreted SmVALs closely related to SmVAL4 (e.g. SmVAL10 and SmVAL18) (Figure S4) in this model of airway inflammation could help the identification and mapping of such IgE epitopes.

After the sequencing and assembly of the *Schistosoma* genome, efforts in schistosome research should shift from the simple identification of genes to the characterization of their functions and interactions with host cells. From this perspective, data presented here should be taken as a first insight on possible functions for members of the SmVAL family. They could have an immunomodulatory role that may be important during parasite penetration. One could hypothesize that SmVAL4 would be involved in the recruitment of mast cells and basophils, inducing secretion of histamine, which could facilitate parasite invasion through vessel dilatation. Additional studies, such as *in situ* hybridization and the evaluation of native proteins should further elucidate the localization and the role of these proteins.

Finally, we believe that, although the airway inflammation model explored here presents some divergences from physiological conditions, it reveals and differentiates the allergic properties of molecules, proving to be useful for studying molecules with allergic potential. In selecting SmVAL molecules to be further investigated as vaccine candidates, we can eliminate those that display allergic potential in this model, such as SmVAL4.

## Supporting Information

Figure S1 **Summary of some SmVALs developmental stage associated mRNA and proteins based on recent data from** ***S. mansoni*** **and** ***S. japonicum*** **.** (A) Upper panel: the developmental stages of the life cycle of schistosomes; (B) SmVALs (mRNA or protein) expressed in these developmental stages. The arrow (↑) indicates up-regulation in a particular stage by Real-time RT-PCR [5], [42] or Microarray data [43], [44], [45]; (•) Identification of the correspondent SmVAL protein or (•^j^) SjVAL by proteomics data [6], [7], [8], [11], [12], [13], [14]. M and F – male and female adult worms, * previously reported on the *Schistosoma* transcriptome analysis [4], (panel (A) was extracted and modified from [4]).
(PDF)

Figure S2 **Phylogenetic analysis of** ***S. mansoni*** **and** ***S. japonicum*** **VALs protein family, demonstrating that SjVAL (AAW27353.1) branches with SmVAL26/28 and 27.** Phylogenetic trees were inferred by ClustalX 1.83 and illustrated by Treeview as described in Methods. The *S. japonicum* protein GenBank accession numbers are indicated in the tree, whereas the *S. mansoni* are the following: SmVAL1 (AAY43180.1), SmVAL2 (XP_002571733.1), SmVAL3 (AAZ04923.2), SmVAL4 (XP_002571676.1), SmVAL5 (ABB88846.2), SmVAL6 (AAY28955.1), SmVAL7 (AAZ04924.1), SmVAL8 (ABW98681.1), SmVAL9 (XP_002582201.1), SmVAL10 (ABO09814.2), SmVAL11 (ABA54555.1), SmVAL12 (XP_002571731.1), SmVAL13 (ABB88843.1), SmVAL14 (XP_002569793.1), SmVAL15 (XP_002582174.1), SmVAL16 (XP_002571817.1), SmVAL17 (XP_002578833.1), SmVAL18 (XP_002571658.1), SmVAL19 (XP_002571657.1), SmVAL20 (CAZ28636.1), SmVAL21 (XP_002578075.1), SmVAL22 (XP_002574629.1), SmVAL23 (XP_002582175.1), SmVAL24 (XP_002574962.1), SmVAL25 (XP_002574963.1), SmVAL26 (XP_002577262.1), SmVAL27 (XP_002577271.1), SmVAL28 (XP_002582199.1) and SmVAL29 (XP_002571340.1).
(PDF)

Figure S3 **Alignment of the derived amino acid sequence of SmVAL26 and SjVAL26 (AAW27353.1).** Highlighted are the SCP domain (continuous box). The regions with high identity and similarity between sequences are shown as black and gray columns, according to the Clustal X algorithm.
(PDF)

Figure S4 **Alignment of the derived amino acid sequence of SmVAL4, 5, 10, 18, 26, 27 and 28.** Demonstration that SmVAL4, 10 and 18 (all secreted during the cercaria-schistosomulum transformation process) are more closely related, whereas SmVAL5, 26, 27 and 28 (all detected in the egg stage) are more related to each other. The regions with high identity and similarity between SmVALs are shown as black and gray columns, according to the Clustal X algorithm.
(PDF)

Table S1 **Synthetic genes used in this study.** ^a^Redesigned sequence using DNA2.0 codon optimization algorithms for expression in *Pichia pastoris*.
(PDF)
