# Supplementary figures and images for: Schistosoma mansoni Venom Allergen Like Proteins Present Differential Allergic Responses in a Murine Model of Airway Inflammation
Source: PLoS Negl Trop Dis. 2012 Feb 7;6(2):e1510. doi: 10.1371/journal.pntd.0001510 (PMC3274501; doi:10.1371/journal.pntd.0001510)

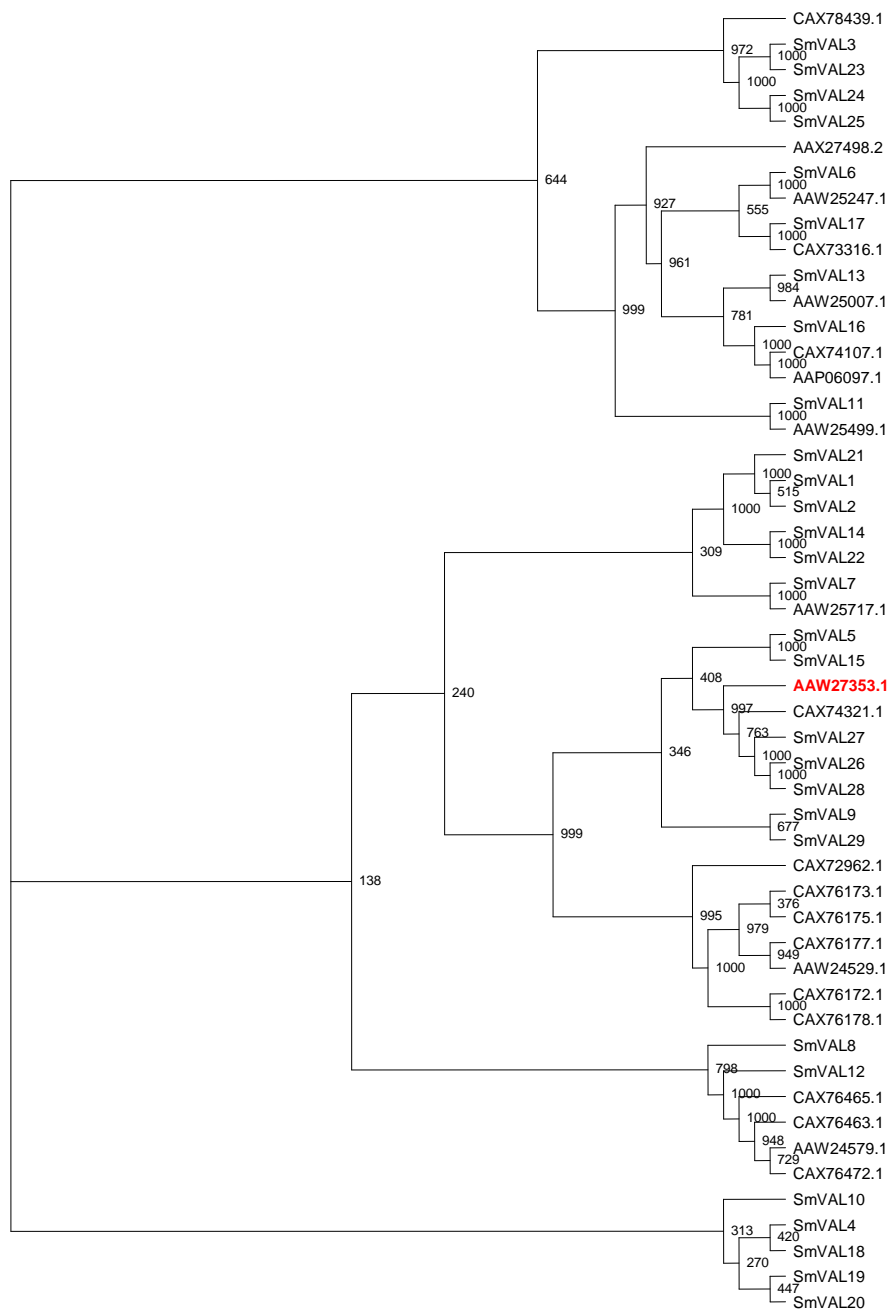

Supplement: Figure S2 — Phylogenetic analysis of S. mansoni and S. japonicum VALs protein family, demonstrating that SjVAL (AAW27353.1) branches with SmVAL26/28 and 27. Phylogenetic trees were inferred by ClustalX 1.83 and illustrated by Treeview as described in Methods. The S. japonicum protein GenBank accession numbers are indicated in the tree, whereas the S. mansoni are the following: SmVAL1 (AAY43180.1), SmVAL2 (XP_002571733.1), SmVAL3 (AAZ04923.2), SmVAL4 (XP_002571676.1), SmVAL5 (ABB88846.2), SmVAL6 (AAY28955.1), SmVAL7 (AAZ04924.1), SmVAL8 (ABW98681.1), SmVAL9 (XP_002582201.1), SmVAL10 (ABO09814.2), SmVAL11 (ABA54555.1), SmVAL12 (XP_002571731.1), SmVAL13 (ABB88843.1), SmVAL14 (XP_002569793.1), SmVAL15 (XP_002582174.1), SmVAL16 (XP_002571817.1), SmVAL17 (XP_002578833.1), SmVAL18 (XP_002571658.1), SmVAL19 (XP_002571657.1), SmVAL20 (CAZ28636.1), SmVAL21 (XP_002578075.1), SmVAL22 (XP_002574629.1), SmVAL23 (XP_002582175.1), SmVAL24 (XP_002574962.1), SmVAL25 (XP_002574963.1), SmVAL26 (XP_002577262.1), SmVAL27 (XP_002577271.1), SmVAL28 (XP_002582199.1) and SmVAL29 (XP_002571340.1). (PDF) [file pntd.0001510.s002.pdf]
