# Supplementary material for: Schistosoma mansoni Venom Allergen Like Proteins Present Differential Allergic Responses in a Murine Model of Airway Inflammation
Source: PLoS Negl Trop Dis. 2012 Feb 7;6(2):e1510. doi: 10.1371/journal.pntd.0001510 (PMC3274501; doi:10.1371/journal.pntd.0001510)
Supplement: Figure S3 — Alignment of the derived amino acid sequence of SmVAL26 and SjVAL26 (AAW27353.1). Highlighted are the SCP domain (continuous box). The regions with high identity and similarity between sequences are shown as black and gray columns, according to the Clustal X algorithm. (PDF) [file pntd.0001510.s003.pdf]

```
SmVal26 : KLDDAMRNELLTLHNEARQAVRNGQLFGQPIAVSIKPLKWNVELERKAQILSDQC :  55
SjVal26 : VMDNRMRTQLLSLHNTARAAVRNGQLSGQPIAVSMKLLKWNTELEMKAQFLSDQC :  55
            D  MR  LL LHN AR AVRNGQL GQPIAVS K LKWN ELE KAQ LSDQC

SmVal26 : RVGHDTNADRQIPEFQYVGQNWAGATDIKTGFQLWLDEYNNYDFYTRTCRMGQCG : 110
SjVal26 : RFGHDTNNDRKTSQFPYVGQNWAGSQDIETGFQLWLDEYKYYDFNTGTCHLAQCG : 110
          R GHDTN DR    F YVGQNWAG  DI TGFQLWLDEY  YDF T TC   QCG

SmVal26 : HYTQLVWEDTTDVGCGVTDCPNFPYGLSIVCNYGPG-------------HITDMK : 152
SjVal26 : HYTQIVWENTTDIGCGVSNCPNIPYKLSIVCNYGPAGNHIGQAPYKTATGVTDSE : 165
          HYTQ VWE TTD GCGV  CPN PY LSIVCNYGP                TD

SmVal26 : ------------------------------------------------------- :   -
SjVal26 : PQQQQTKQTVTSGYNSNNNSSNNNSNNNTSSNNNTSDISDNNSSNSNSNNDIINN : 220

SmVal26 : ------------------------------------------------------- :   -
SjVal26 : NNNNNNNNNNGNSNNDINSINNNISSSTSNSDISNNNDNNTDNDNSSNNIKNEGA : 275

SmVal26 : ------------------------------------------------------- :   -
SjVal26 : EGEKYYLDGYNNRKSKCNRKSRQTYNKPITNNQLPSPSLCSSNSNSYYGSPKLIP : 330
```
