# Supplementary material for: Schistosoma mansoni Venom Allergen Like Proteins Present Differential Allergic Responses in a Murine Model of Airway Inflammation
Source: PLoS Negl Trop Dis. 2012 Feb 7;6(2):e1510. doi: 10.1371/journal.pntd.0001510 (PMC3274501; doi:10.1371/journal.pntd.0001510)
Supplement: Figure S4 — Alignment of the derived amino acid sequence of SmVAL4, 5, 10, 18, 26, 27 and 28. Demonstration that SmVAL4, 10 and 18 (all secreted during the cercaria-schistosomulum transformation process) are more closely related, whereas SmVAL5, 26, 27 and 28 (all detected in the egg stage) are more related to each other. The regions with high identity and similarity between SmVALs are shown as black and gray columns, according to the Clustal X algorithm. (PDF) [file pntd.0001510.s004.pdf]

```
SmVAL4  : ---KLSEGQRAIYNFHKKVRKDVKNCRIPGQPPAKNLTKLKWNKLLANKAKQQAKRCKYDSNDPNDFIIGDFESIGQNLADYPTIEGAMKDWLEEYKNYNFEKNQCNG-DCKN : 109
SmVAL18 : KKLKLPKEVREVFQLHKYYRNSIRFCQMPKQPPAKYMSKLQWNKHLAEKAQLTASRCDYSYDSPSDMRFEEFSSVAQNIADSPTIEKAVASWFIEYKNYSFDDNTCKD-TCMQ : 112
SmVAL10 : ---DKSSTKELIFNFHNKIREDVFKGVLSGQPKAKKMSKLKWNKLLAKLAKGHVQKCILDSGDLGKLYVGKFDSVGQTVAEHTSIQNILDTWLEEKNDYDLDKNTCEN-ECGN : 109
SmVAL26 : --KLDDAMRNELLTLHNEARQAVRNGQLFGQPIAVSIKPLKWNVELERKAQILSDQCRVGHDTNADRQIPEFQYVGQNWAGATDIKTGFQLWLDEYNNYDFYTRTCRMGQCGH : 111
SmVAL28 : --KLDDAMRNELLTLHNEARQAVRNGQLFGQPIAVSIKPLKWNVELERKAQILSDQCRVGHDTNADRQIPEFQYVGQNWAGATDIKTGFQLWLDEYKNYDFYTRTCRMGQCGH : 111
SmVAL27 : --KLDDTMRNELLTLHNKARQSVRNGQLFGQPRAVSIKPLQWNVELERKAQNLSDRCQVGHDTNADRKIPKFQYVGQNWAGAKDIKTGFQSWLDEYKNYDFYTRTCRMGQCGH : 111
SmVAL5  : --AMDNATREKLLKLHNNARVSVMHGRLEGQPIAKSIKPLKWNMELEKKAQMLADTCYFGPDSAIERKVPGFTNVGQNWAGASTVDIGFQRWLNEYKNYDFFNRLCLVGRCIH : 111
                         H   R  v      gQP A     L WN  L  kA      C              F  vgQn A    i      Wl Ey nY f    C    C

SmVAL4  : YKQMVWNTTEEIGCGYEKCGKN---YLIVCNYAPGDSEDR---------------------------------------------PYEAKPESKCNKSE------------- : 160
SmVAL18 : YKQMVKGEETEIGCGVQKCSNR---FLVVCNYSPAAEEDKQ--------------------------------------------PYEKGTQENCDDVDDAEY--------- : 168
SmVAL10 : YKQLVWANTTDIGCASNKCGNR---YMVVCNYAPGADDER---------------------------------------------PYEKDS--------------------- : 152
SmVAL26 : YTQLVWEDTTDVGCGVTDCPNFPYGLSIVCNYGPGGNYP--------------------------------------------GRPLYRTT--------------------- : 158
SmVAL28 : YTQLVWEDTTDVGCGVTDCPNFPYGLSIVCNYGPGGNYP--------------------------------------------GRPLYRTT--------------------- : 158
SmVAL27 : YTQLVWEDTTDVGCGVTKCPNFPYGLSIVCNYGPGGNYA--------------------------------------------GRPLYRTT--------------------- : 158
SmVAL5  : YTQIVWENTTDIGCGVATCPHSPFKLSIVCNYGPGGGCPRQFPYSVKGLYRQWTIKYGRKWYSWRYGRRCRPVYVKQRCNHTNERQLNRTPAIQKKRYKLIKQYLCPNKQKV : 223
          Y Q Vw  tt  GCg   C          VCNY Pg                                                 p
```

Cercaria - Schistosomulum transformation (SmVAL4, SmVAL18, SmVAL10)

Egg (SmVAL26, SmVAL28, SmVAL27, SmVAL5)
