# Supplementary material for: Schistosoma mansoni Venom Allergen Like Proteins Present Differential Allergic Responses in a Murine Model of Airway Inflammation
Source: PLoS Negl Trop Dis. 2012 Feb 7;6(2):e1510. doi: 10.1371/journal.pntd.0001510 (PMC3274501; doi:10.1371/journal.pntd.0001510)
Supplement: Table S1 — Synthetic genes used in this study. aRedesigned sequence using DNA2.0 codon optimization algorithms for expression in Pichia pastoris. (PDF) [file pntd.0001510.s005.pdf]

Table S1. Synthetic genes used in this study.

	Synthetic Genes[a]
SmVAL4	GAATTCAAGCTTAGTGAAGGTCAACGTGCTATTTACAACTTCCATAAAAAGGTGAGAAAAGACGTAAAGAAT<br>TGTAGAATACCAGGACAACCACCTGCAAAGAACCTTACCAAACTGAAATGGAACAAACTTCTGGCAAATAAG<br>GCAAAACAACAAGCTAAAAGATGTAAGTATGACTCCAATGATCCAAACGATTTCATTATCGGAGATTTTGAG<br>TCAATAGGACAAAATCTAGCCGACTACCCAACCATCGAAGGCGCTATGAAGGACTGGTTGGAGGAGTACAAA<br>AATTACAACTTTGAGAAAAACCAGTGTAACGGAGACTGTAAGAATTATAAGCAAATGGTGTGGAATACTACT<br>GAAGAAATAGGCTGTGGTTATGAGAAGTGCGGGAAAAATTACTTGATCGTTTGTAACTACGCCCCTGGTGAT<br>TCAGAAGATAGACCATACGAAGCCAAACCAGAATCTAAGTGTAACAAATCCGAATCTAGA
SmVAL26	GAATTCAAGCTTGATGATGCCATGAGAAATGAGTTACTTACACTACACAATGAGGCAAGACAGGCTGTTAGA<br>AATGGACAACTGTTTGGCCAACCAATCGCCGTTAGTATTAAACCATTAAAATGGAATGTTGAGCTAGAGAGA<br>AAAGCACAGATCTTATCAGATCAATGCAGAGTAGGTCACGACACAAACGCCGATCGTCAGATACCTGAGTTC<br>CAGTACGTTGGTCAAAATTGGGCTGGAGCAACTGATATCAAAACCGGCTTTCAGTTGTGGCTTGATGAGTAT<br>AATAACTACGACTTTTATACAAGAACCTGTAGAATGGGCCAATGCGGGCACTACACTCAATTGGTTTGGGAA<br>GACACCACCGACGTGGGATGTGGTGTTACAGATTGTCCAAATTTTCCTTACGGTTTGTCAATCGTTTGTAAC<br>TACGGTCCAGGCCACATCACGGACATGAAGTCTAGA

[a]Redesigned sequence using DNA2.0 codon optimization algorithms for expression in *Pichia pastoris.*
