# Supplementary material for: Schistosoma mansoni Venom Allergen Like Proteins Present Differential Allergic Responses in a Murine Model of Airway Inflammation
Source: PLoS Negl Trop Dis. 2012 Feb 7;6(2):e1510. doi: 10.1371/journal.pntd.0001510 (PMC3274501; doi:10.1371/journal.pntd.0001510)
Supplement: Figure S1 — Summary of some SmVALs developmental stage associated mRNA and proteins based on recent data from S. mansoni and S. japonicum . (A) Upper panel: the developmental stages of the life cycle of schistosomes; (B) SmVALs (mRNA or protein) expressed in these developmental stages. The arrow (↑) indicates up-regulation in a particular stage by Real-time RT-PCR [5], [42] or Microarray data [43], [44], [45]; (•) Identification of the correspondent SmVAL protein or (•j) SjVAL by proteomics data [6], [7], [8], [11], [12], [13], [14]. M and F – male and female adult worms, * previously reported on the Schistosoma transcriptome analysis [4], (panel (A) was extracted and modified from [4]). (PDF) [file pntd.0001510.s001.pdf]

(A)

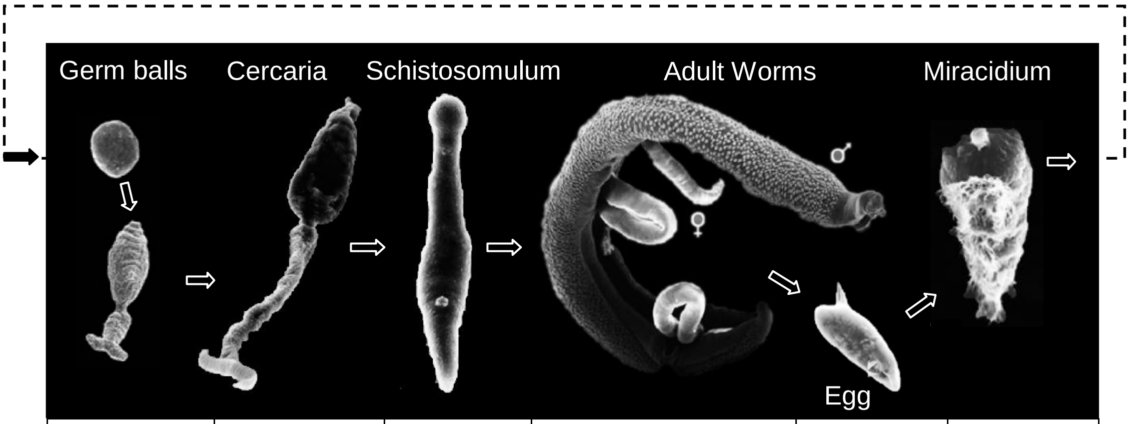

(B)

	Germ balls RNA	Germ balls Prot	Cercaria RNA	Cercaria Prot	Schistosomulum RNA	Schistosomulum Prot	Adult Worms RNA	Adult Worms Prot	Egg RNA	Egg Prot	Miracidium RNA	Miracidium Prot
SmVAL24	↑											
SmVAL25	↑											
SmVAL4*	↑		↑	•								
SmVAL18	↑			•								
SmVAL10			↑	•								
SmVAL1			↑									
SmVAL16			↑									
SmVAL17			↑									
SmVAL21			↑									
SmVAL7			↑		↑		↑[M]					
SmVAL6			↑				↑[M]	•				
SmVAL13					↑		↑[F]					
SmVAL8							↑					
SmVAL12							↑					
SmVAL26						•[j]				•		•
SmVAL5*										•	↑	•
SmVAL11											↑	
SmVAL2*										•	↑	•
SmVAL3*										•	↑	•
SmVAL9										•	↑	•
SmVAL28												•
SmVAL15												•
SmVAL23												•
SmVAL27												•
